# Cognitive impairment and frailty in depressed elderly

**DOI:** 10.1192/j.eurpsy.2022.394

**Published:** 2022-09-01

**Authors:** M. Arts, S. Petrykiv, L. Jonge

**Affiliations:** GGZWNB, Psychiatry, Bergen op Zoom, Netherlands

**Keywords:** cognition, Older Adults, Frailty, Depression

## Abstract

**Introduction:**

Cognitive frailty has recently been defined as the co-occurrence of physical frailty and cognitive impairment. Late-life depression (LLD) is associated with both physical frailty and cognitive impairment, especially processing speed and executive functioning.

**Objectives:**

The objective of this study was to investigate the association between physical frailty and cognitive functioning in depressed older persons.

**Methods:**

A total of 378 patients (>60 years) with depression according to DSM-IV criteria and a MMSE score of 24 points or higher were included. The physical frailty phenotype was examined as well as its individual criteria (weight loss, weakness, exhaustion, slowness, low activity). Cognitive functioning was examined in 4 domains: verbal memory, working memory, interference control, and processing speed.

**Results:**

Of the 378 depressed patients (range 60-90 years; 66.1% women), 61 were classified as robust (no frailty criteria present), 214 as prefrail (1 or 2 frailty criteria present), and 103 as frail (>3 criteria). Linear regression analyses, adjusted for confounders, showed that the severity of physical frailty was associated with poorer verbal memory, slower processing speed, and decreased working memory, but not with changes in interference control.

**Conclusions:**

Physical frailty in LLD is associated with poorer cognitive functioning, although not consistently for executive functioning. Future studies should examine whether cognitive impairment in the presence of physical frailty belongs to cognitive frailty and is indeed an important concept to identify a specific subgroup of depressed older patients, who need multimodal treatment strategies integrating physical, cognitive, and psychological functioning.

**Disclosure:**

No significant relationships.

